# Plasma Membrane Transporters in Modern Liver Pharmacology

**DOI:** 10.6064/2012/428139

**Published:** 2012-10-14

**Authors:** Jose J. G. Marin

**Affiliations:** ^1^Laboratory of Experimental Hepatology and Drug Targeting (HEVEFARM), IBSAL, University of Salamanca and CIBERehd, Spain; ^2^Department of Physiology and Pharmacology, Campus Miguel de Unamuno E.D. S09, 37007 Salamanca, Spain

## Abstract

The liver plays a crucial role in the detoxification of drugs used in the treatment of many diseases. The liver itself is the target for drugs aimed to modify its function or to treat infections and tumours affecting this organ. Both detoxification and pharmacological processes occurring in the liver require the uptake of the drug by hepatic cells and, in some cases, the elimination into bile. These steps have been classified as detoxification phase 0 and phase III, respectively. Since most drugs cannot cross the plasma membrane by simple diffusion, the involvement of transporters is mandatory. Several members of the superfamilies of solute carriers (SLC) and ATP-binding cassette (ABC) proteins, with a minor participation of other families of transporters, account for the uptake and efflux, respectively, of endobiotic and xenobiotic compounds across the basolateral and apical membranes of hepatocytes and cholangiocytes. These transporters are also involved in the sensitivity and refractoriness to the pharmacological treatment of liver tumours. An additional interesting aspect of the role of plasma membrane transporters in liver pharmacology regards the promiscuity of many of these carriers, which accounts for a variety of drug-drug, endogenous substances-drug and food components-drug interactions with clinical relevance.

## 1. Introduction

**Figure 1:**
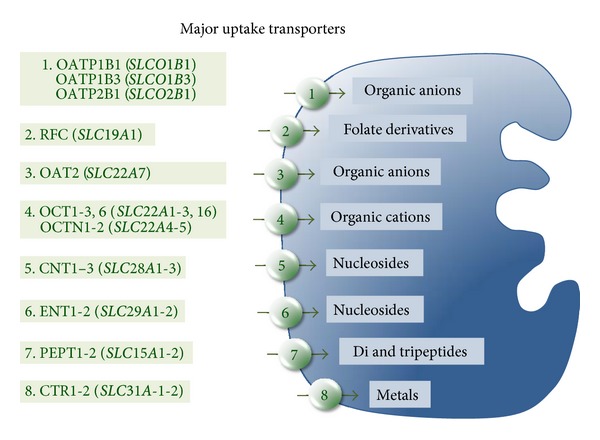
Major transporters involved in drug uptake by hepatocytes.

Few drugs with very different chemical structure, but with the shared characteristic of high lipophilicity, can enter the cells by simple diffusion across the lipid bilayer of the plasma membrane. This is not, however, the common rule. Owing to the fact that the majority of drugs are polar compounds, the participation in their uptake of plasma membrane transporters, belonging to the solute carrier (SLC) superfamily, is required. This includes approximately 300 genes classified into 43 families [1]. Owing to the large variety of carriers involved in transport processes, either by facilitated diffusion or by secondary active symport or antiport concentrative mechanisms, and to their marked overlap in substrate specificity, at the plasma membrane of hepatocytes there is a gate for the uptake of almost every drug [2]. The presence in the liver cells of the required transporter, mainly at the basolateral or sinusoidal membrane of hepatocytes (Figure 1), and its level of expression under pathological circumstances, when the drug is needed, determine the bioavailability and hence the efficacy of the pharmacological agent.

**Figure 2:**
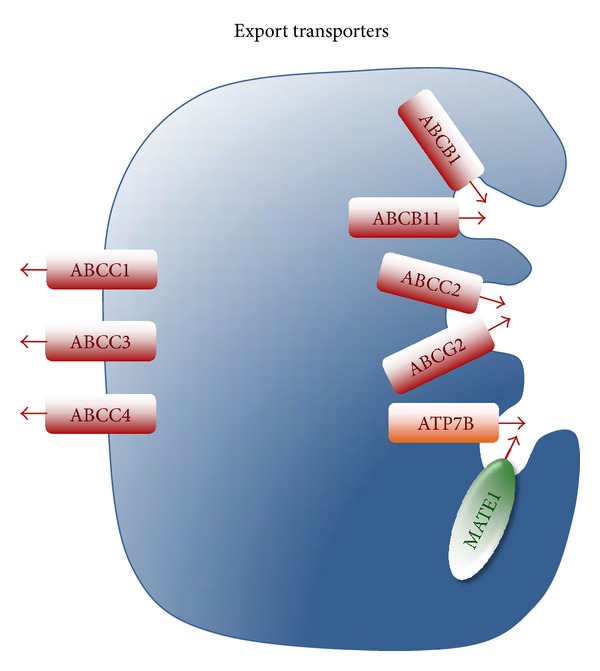
Export pumps involved in drug export across the basolateral and canalicular membrane of hepatocytes.

Regarding the overall detoxification process, and when the desired therapeutic action must take place in the liver itself, the ability to take up the drug and the function of export pumps accounting for the active efflux toward the bile or back to blood are similarly important. These pumps are primary active transporters able to use metabolic energy in the form of ATP to carry out the export of a large variety of substrates across the basolateral and the apical or canalicular plasma membrane of hepatocytes and cholangiocytes. Most of these transporters, but not all, belong to the superfamily of ATP-binding cassette (ABC) proteins that in humans includes 48 genes classified into 7 different families (from ABCA to ABCG). Under physiological circumstances many of them play an important role in barrier mechanisms and secretory functions. Thus, in hepatocytes ABC proteins located at the canalicular membrane are involved in bile formation (Figure 2). Accordingly, impairment in the expression or function of these pumps accounts for several forms of inherited or acquired intrahepatic cholestasis [3]. 

Both uptake and efflux transporters expressed in small intestine, liver, and kidney, either in healthy tissues or in tumours, are involved in the mechanisms that determine the pharmacological efficacy of many drugs, including antitumour agents. Thus, a reduction in the uptake of cytostatic drugs has been included among the mechanisms of chemoresistance type 1a (MOC-1a) [4]. Changes in the expression of uptake transporters or the existence of genetic variants [5], as well as the simultaneous administration of inhibitors, may greatly affect (i) the appearance of adverse reactions due to drug-induced toxicity; (ii) the ability of the body to carry out the absorption or excretion of a certain drug; (iii) the efficacy of this drug to reach sufficient intracellular concentrations in the target cells [6]. At this respect, a question of great impact and increasing interest is the possibility that coadministration of certain drugs with food containing substrates or inhibitors of the involved transporter may affect the overall pharmacokinetics of these drugs. However, our knowledge of the potential risk of nutrient-drug interactions is still limited [7].

The net transport function is also determined by the existence of mechanisms of internalization of carrier proteins from the plasma membrane. Interestingly, this process can be pharmacologically manipulated. A good example is the effect of 4-phenylbutyrate, a drug used to treat ornithine transcarbamylase deficiency, on the amount of the bile salt export pump (BSEP, gene symbol *ABCB11*) protein inserted at the canalicular membrane of hepatocytes. Treatment with 4-phenylbutyrate induces downregulation of subunits (*α*- and *μ*2-adaptin) of the AP2 adaptor complex. This mediates clathrin-dependent endocytosis and subsequent degradation of BSEP [8].

## 2. Drug Uptake Transporters

In addition to changes in the expression levels due to exogenous or endogenous factors that may be influenced by different stimuli, such as seasonal, endocrine, nutritional, and exposure to xenobiotics, the existence of polymorphisms in genes of SLC superfamily expressed in liver cells accounts for a marked interindividual sensitivity to many drugs whose activity is performed on liver cells or whose metabolism and/or elimination depends on the hepatic function [9]. Moreover, it should be taken into account that, although we will focus this paper on liver processes, the availability of the drugs reaching the liver could be greatly modified by similar transporters involved in their intestinal absorption and renal elimination. 

### 2.1. OATP Family

An important role in drug uptake by the liver is played by members of the superfamily of organic anion transporting polypeptides (OATP) encoded by *SLCO* (previously known as *SLC21*) genes [10]. The 11 human isoforms of OATPs are classified into 6 families (from OATP1 to OATP6) on the basis of their amino acid sequence similarities (>40%), and several subfamilies (1A, 1B, etc.) with members sharing higher (>60%) amino acid identity. These proteins have the common characteristic of containing 12 transmembrane domains with both amino and carboxyl termini intracellularly oriented. Three major OATP isoforms are expressed at the basolateral membrane of hepatocytes: OATP1B1 (previously known as OATP-C, gene symbol *SLCO1B1*), OATP1B3 (previously known as OATP8, gene symbol *SLCO1B3*), and OATP2B1 (previously known as OATP-B, gene symbol *SLCO2B1*). However, other members of this family are also expressed in liver (OATP3A1 > OATP1A2 > OATP1C1 > OATP2A1 *≈* OATP4A1) [11]. Neutral anion exchange has been proposed as the general mechanism for most members of this family of transporters, being bicarbonate the exchanged anion [12]. However, recent results indicate that at least the two OATP isoforms primarily expressed in the liver, namely, OATP1B1 and OATP1B3, are electrogenic transporters, although their activity may be strongly affected under circumstances of abnormal variations of local pH [13]. This is pharmacologically important because tumour environment is often acidic and both transporters can be the gate for the entrance in liver tumours of many cytostatic drugs [14], such as irinotecan and its active metabolite, 7-ethyl-10-hydroxycamptothecin (SN-38) [15], flavopiridol [16], methotrexate [17], paclitaxel [18], and several bile acid-cisplatin conjugates (BAMET) [19]. OATP2B1 is expressed in territories other than liver, where this transporter is involved in the uptake of different drugs. Thus, OATP2B1 has been recently identified as a carrier for the antiarrhythmic agent amiodarone [20] and antifolate drugs [21]. Another member of this family, OATP1A2 (previously known as OATP-A, gene symbol *SLCO1A2*), is also expressed, although at low level, in the liver. This transporter is pharmacologically interesting due to its presence in cholangiocytes [22] and because its ability to transport antitumour drugs, such as methotrexate [23] and imatinib [24]. A complete list of substrates for OATP1B1, OATP1B3, and OATP1A2 is shown in Table 1.

Clinically important drug interactions leading to adverse drug reactions have been traditionally associated with impaired metabolic detoxification (phase I and phase II processes) of the administered drug. However, at present the accepted concept includes that some drug interactions result from changes in the activity and/or expression of drug transporters [25]. Owing to the promiscuity of OATP isoforms, there are many examples of drug-drug, endogenous compounds-drug and food components-drug interactions involving these transporters. Thus OATP1A2-mediated uptake of the histamine H1-receptor antagonist fexofenadine, used for the temporary relief of runny nose, sneezing, and nasal stuffiness from common cold, is inhibited by several drugs, including antivirals, antifungals and anticholesterol drugs [26], and flavonoids contained in grapefruit, such as naringin [27]. The ability of grapefruit and orange juices to interfere with OATP-mediated drug uptake has been also suggested for OATP2B1 [28]. Several important drug interactions with clinical relevance have been described for OATP1B1 and OATP1B3, such as cerivastatin-gemfibrozil [29, 30] and atorvastatin-rifampin [31].

The importance of OATPs in the liver handling of many drugs accounts for the fact that environmental and genetic factors able to perturb their expression and activity can contribute to adverse drug reactions (ADR) [32]. This may occur due to single nucleotide polymorphisms (SNPs) that change OATP activity and to epigenetic regulation that modify OATP expression levels, and sometimes these changes accompany certain liver diseases. In patients with primary biliary cirrhosis, haemochromatosis and nonalcoholic steatohepatitis with altered hepatic expression of vitamin C transporters SLC23A1 and SLC23A2, the mRNA levels of OATP1A2, OATP1B1, and OATP1B3 were not changed [33]. In contrast, decreased expression of OATP1B1 has been found as a shared characteristic of hepatocellular carcinoma, and cholangiocarcinoma [34].

Regarding the effect of genetic variants two examples can illustrate the clinical importance of identifying patients carrying polymorphisms that can affect OATP function. This is the case of some SNPs, such as c.521T > C (p.Val174Ala) in *SLCO1B1*, which results in impaired OATP1B1 function [35]. This may account for either adverse or protective effects, as will be commented below. Patients with this SNP show more than 3-fold increased serum concentrations of simvastatin acid, the active metabolite of simvastatin, when receiving this drug [36]. This has been associated with a higher risk of suffering from myopathy when these patients are treated with a high dose of simvastatin (80 mg/day) for some time [37]. In contrast, they are partly protected against the adverse effects of mycophenolic acid [38]. This is the active moiety of mycophenolate mofetil, a drug used as immunosuppressant in patients undergoing kidney transplantation. Unfortunately ADR, characterized by nausea, vomiting, hematotoxicity, and infections, affects up to 50% of treated patients. Interestingly, mycophenolic acid is not a substrate of OATP1B1, whereas its glucuronate derivatives are taken up by hepatocytes through this carrier [39]. Thus, mycophenolic acid is biotransformed by hepatocytes to glucuronides that are secreted into bile through the canalicular multidrug resistance-associated protein (MRP2, gene symbol *ABCC2*). The reabsorbed metabolites are maintained in the enterohepatic circulation due in part to OATP1B1 activity. This recycling is impaired in carriers of *SLCO1B1*-c.521C variant in whom reduced hepatocellular uptake probably results in enhanced renal elimination of polar mycophenolic acid, which reduces the risk of suffering from mycophenolate mofetil-induced ADR [6]. 

In addition to pharmacokinetics, OATP-mediated drug interactions can also have an important impact in toxicokinetics. Thus, phalloidin causes severe liver damage characterized by marked cholestasis, which is due in part to irreversible polymerization of actin filaments. Hepatocyte uptake of phalloidin is carried out mainly by OATP1B1. This process can be inhibited by compounds, such as BALU-1, a nontoxic bile acid derivative able to reduce the uptake of the toxin without impairing endogenous bile acid uptake by the major carrier accounting for this process, that is, the sodium-taurocholate cotransporting polypeptide (NTCP, gene symbol *SLC10A1*) [40]. In a rat model of phalloidin-induced hepatotoxicity, BALU-1 was able to protect against liver injury, due in part to the inhibition of phalloidin liver uptake and an enhancement in the biliary secretion of the toxin [41].

### 2.2. RFC Family

Folate is a member of vitamin B group and is required for the transfer of one carbon unit during nucleic acid synthesis and for amino acid metabolism. Under physiological conditions folic acid is taken up by the proton-coupled folate transporter (PCFT, gene symbol *SLC46A1*) and the reduced folate carrier (RFC, gene symbol *SLC19A1*) [42]. The latter, which is expressed in liver cells, is also the gate of entrance in the cell of antifolate agents, such as methotrexate [43]. Genetic variants in this gene have been associated to the lack of efficiency of methotrexate in several types of tumours [44, 45].

### 2.3. OAT Family

Organic anion transporters (OAT) are believed to behave as anion exchangers. They are able to transport many different drugs [46]. The preferred substrates are anionic compounds with two carboxylate groups. The first member of the family identified was OAT1 (gene symbol *SLC22A6*), which is mainly expressed in kidney. In human liver although several isoforms have been detected OAT2 (*SLC22A7*) is largely the major one [11, 47]. Several classes of drugs interact with human OATs [48]. These include antiviral drugs, ACE inhibitors, angiotensin II receptor antagonists, diuretics, HMG-CoA reductase inhibitors, *β*-lactam antibiotics, uricosuric drugs, antitumour drugs, such as methotrexate [47], 5-fluorouracil and paclitaxel [49], and oral selective estrogen receptor modulators, such as raloxifene [50], which has estrogenic actions on bone and antiestrogenic effects on uterus and breast. This accounts for its use in the prevention of osteoporosis in postmenopausal women and as part of adjuvant chemotherapy in breast cancer treatment. Identified potential substrates of OAT2 with pharmacological interest are listed in Table 2.

### 2.4. OCT/OCTN Family

Although belonging to the same family of genes, that is, *SLC22A*, the substrates and mechanisms of transport of organic cations transporters (OCT) are different to these of OATs. Thus, OCTs are sodium-independent electrogenic carriers able to transport small organic cations (type I), such as tetraethylammonium [51]. The predicted membrane topology of these transporters consists of 12 transmembrane domains with intracellular amino and carboxyl termini [52]. Three members of this family have been described in humans, OCT1, which is primarily expressed in liver and to a lesser extent in other organs, OCT2 and OCT3, also expressed in liver (*SLC22A1, SLC22A2,* and *SLC22A3*, resp.). The high abundance of OCT1 at the sinusoidal membrane of hepatocytes accounts for the importance of this carrier in the handling of cationic drugs by the liver (Table 2). An important example among these drugs is metformin, a biguanide developed from galegine, a guanidine derivative found in *Galega officinalis*, which is widely used for the treatment of type 2 diabetes mellitus. The oral absorption, hepatic uptake, and renal excretion of metformin are mainly mediated by OCT isoforms. Thus, variability in the expression of OCT1 and OCT3 in liver, and hence presumably in the liver capability to take up metformin, has been suggested to play an important role in the interindividual differences found in the clinical efficacy of this drug [53]. Interestingly, an intron variant (G > A, SNP rs2289669) of the multidrug and toxin extrusion transporter-1 (MATE1, gene symbol *SLC47A1*), mainly expressed in the kidney, but also at the canalicular membrane of hepatocytes [54] (Figure 2), has also been associated with a small increase in the antihyperglycemic effect of metformin in patients carrying this genetic variant [53]. 

Members of the family of carnitine/organic cation transporter (OCTN) are able to transport cations with high molecular weight (type II cations) and carnitine in a sodium-dependent and -independent manner. The transporter of carnitine and organic cations OCT6 (*SLC22A16*) also belongs to this group of carriers. The member of this family of transporters most abundantly expressed in liver cells is OCTN1, which behaves as an organic cation uniporter or H^+^/organic cation antiporter able to translocate its substrates in both directions. In addition to its presence at the plasma membrane, OCTN1 has been also localized in mitochondria [55], which may have important physiological and pharmacological implications. 

Owing to the fact that many of the novel strategies to treat both hepatocellular carcinoma and cholangiocarcinoma include the use of tyrosine kinase inhibitors and many of them are cationic compounds [56], transporters able to transport these compounds are attracting much attention in modern pharmacology of liver cancer. There is already available information regarding the importance of the OCT1-tyrosine kinase inhibitors tandem in tumours affecting extrahepatic tissues. Thus, expression levels of OCT1 have been associated to the response of patients suffering from chronic myeloid leukemia to imatinib, which is taken up by this carrier [57]. Moreover, induction of the expression of OCT1 in lymphoma cells results in enhanced sensitivity to irinotecan and paclitaxel [58].

### 2.5. CNT and ENT Families

Owing to the key role of nucleosides as precursors for nucleotides required in DNA and RNA synthesis, their physiological and pharmacological interest is evident. Nucleosides are taken up by all cells using either sodium-dependent concentrative nucleoside transporters (CNT; gene symbol *SLC28A*) and/or facilitated carriers of the equilibrative nucleoside transporters family (ENT; gene symbol *SLC29A*). Members of both families are able to transport natural nucleosides and many of their derivatives (Table 3). In human liver major CNT isoform is CNT2 (*SLC28A2*), followed by CNT3 (*SLC28A3*) and CNT1 (*SLC28A1*). These have a predicted topology consisting of 13 transmembrane domains with cytoplasmic amino termini and extracellular carboxyl termini [59, 60]. In contrast to CNTs, ENTs are low-affinity equilibrative carriers able to transport their substrates down concentration gradients because nucleosides are intracellularly biotransformed into nucleotides. ENTs consists of 11 transmembrane domains with intracellular amino termini and extracellular carboxyl termini [61, 62]. In human liver three isoforms are expressed in the following order of abundance: ENT1 (*SLC29A1*) > ENT3 (*SLC29A3*) > ENT2 (*SLC29A2*).

Since nucleotides are essential for cell proliferation, many nucleoside derivatives with antiviral and antitumour activity have been synthesized to interfere with the normal use of these compounds by viruses and cancer cells, respectively. Thus, analogues of purine bases such as 6-mercaptopurine and 6-thioguanine, and of pyrimidine bases, such as 5-fluorouracil and gemcitabine constitute an important group of antitumour drugs that require plasma membrane carriers to reach their intracellular molecular targets. Although some of these drugs can be taken up by OATs [49, 63], the main route for this process is through CNTs and/or ENTs. Thus, nucleoside transporters, in particular hENT1, seem to play an important role in predicting clinical outcome after gemcitabine chemotherapy for several types of cancer including cholangiocarcinoma [64]. 

Treatment for hepatitis C virus infection currently consists of pegylated interferon and ribavirin, a nucleoside analogue, which is primarily taken up by ENT1. It has been suggested that reduced expression of this transporter may be involved in the acquired resistance to the treatment of hepatitis C with the drug [65]. Interestingly, infection of liver cells with hepatitis C viruses markedly affects vectorial transport processes typical of healthy hepatocytes [66].

### 2.6. PEPT Family

The family of peptide transporters (PEPT) includes two members, PEPT1 (*SLC15A1*) and PEPT2 (*SLC15A2*); both are highly expressed in liver. Topologically, they are predicted to have 12 transmembrane domains with both termini (amino and carboxyl) located intracellularly [67, 68]. These carriers are able to transport a broad range of substrates, which includes di- and tripeptides but not single amino acids or tetrapeptides (Table 3). The transport process is driven by the inward translocation of protons, which results in an electrogenic balance of charge. The pharmacological importance of PEPTs is based on the fact that several *β*-lactam antibiotics, such as cephalosporines (cefadroxil, cefixime and ceftibuden) and penicillins (amoxicillin and ampicillin), the porphyrin precursor 5-aminolevulinic acid, and several anticancer drugs, such as bestatin, are among the substrates that can be taken up by these transporters [11].

### 2.7. CTR Family

SLC31 family members, together with the Cu-ATPases, are involved in the cellular copper homeostasis. The CTR1 transporter (gene symbol *SLC31A1*) is located at the plasma membrane of many cells, including hepatocytes. This carrier is able to take up monovalent copper by an energy-independent mechanism [69]. In contrast, CTR2 (gene symbol *SLC31A2*) appears to be a vacuolar/vesicular transporter [70]. Functional copper transporters appear to be trimeric with each subunit having three transmembrane regions and an extracellular N-terminus. CTR1 has been suggested to be involved in the transport of cisplatin-related drugs [71]. Indeed, reduction in the expression of this transporter has been described among the phenotypic changes occurring during development of cisplatin chemoresistance in colon cancer cells [72]. CTR2 has also been associated with chemoresistance to cisplatin. Thus, knockdown of CTR2 markedly increases the tumour accumulation of cisplatin and greatly enhances its therapeutic efficacy [73]. The exact mechanism is not known, but recent evidence suggests that CTR2 regulates the transport of cisplatin in part through control of the rate of macropinocytosis via activation of Rac1 and Cdc42 [73].

## 3. Drug Targeting

Owing to the relative or marked selectivity of the expression at the basolateral and apical membranes of hepatocytes and cholangiocytes of carrier proteins involved in the transport of endobiotic and xenobiotic compounds, including many drugs, an important role of these transporters in the development of targeted therapies has been suggested [74]. The usefulness of this approach varies (i) from the possibility of using these transporters in the targeting of drug delivery systems, which can be useful either to direct anticancer drugs towards tumours located in the hepatobiliary system or toward healthy hepatocytes in order to induce or inhibit a metabolic process; (ii) to facilitate the hepatobiliary excretion once the drug has been released from the site of regional administration, for instance, during intraarterial chemoembolization of tumours. This justifies the growing interest in developing novel derivatives of cholephilic organic anions, such as bile acids, with maintained pharmacological effect characteristic of the active agent but with enhanced hepatotropism. These novel targeted pharmacological tools may be useful in the treatment of many different liver diseases, such as in anticancer chemotherapy [56]. 

The interest of using bile acids to these aims is based on the following characteristics: versatile derivatization possibilities, rigid steroidal backbone, enantiomeric purity, availability, and the low cost of natural bile acids for use in chemical reactions. Thus, bile acids are versatile building blocks to which many different substances can be attached at different positions of the steroidal skeleton or on the side chain via different chemical bonds, which can be further varied by linkers with different structures, lengths, stereochemistries, polarities, and/or functional groups. This pharmacological approach has been investigated for the targeting of very different types of drugs [75–79]. 

From the pharmacological point of view, an interesting question regarding drug design is to know the predicted topology of the interaction between the carrier and its substrates because this determines the choice of the site in the Trojan-Horse molecule to be used for its conjugation with the active agent. In the case of bile acids the possibilities for conjugating a drug include hydroxyl groups, in particular the one located at the 3*α*-position, and the carboxyl group on the side chain. The decision will depend on the expected advantages of the resulting drug, that is, enhanced intestinal absorption, improved liver vectoriality, and so forth. 

Transporters of the SLC10A family, such as NTCP in hepatocytes and the apical sodium-dependent bile acid transporter (ASBT, *SLC10A2*) in cells of the intestinal epithelium and cholangiocytes, which are highly efficient in transporting bile acids, have been reported to interact with the region of these molecules that contains its side chain [80]. To use these transporters as uptake gates in a given drug targeting strategy, the bile acid side chain must be maintained in its natural configuration, using other groups, such as the 3*α*-hydroxyl one to bind the active agent. In contrast, derivatives obtained by coupling an active agent to the bile acid side chain are taken up by typically hepatic members of human OATP family [19]. Both strategies have been used to target toward liver tumours either organic [81, 82] or inorganic [83–87] moieties. The latter is particularly interesting because of the small size of the resulting molecule, which would increase the probability of preserving both substrate properties in regards to bile acid transporters and reactivity versus DNA, and hence the antiproliferative effect of these metals, in particular platinum(II) such as in cisplatin [88]. Cytostatic bile acid derivatives, such as BAMETs, are a good example of the versatility of targeted drugs because, although they were first synthesized to enhance their water miscibility [89], this family of compounds has proven to be excellent to target cytostatic agents toward tumours located in tissues of the enterohepatic circuit [74]. Moreover, they have the beneficial characteristic of being efficiently taken up by the liver and eliminated into bile. This reduces the amount of drug that escaping from the tumour, might reach the general circulation during regional therapy [90, 91].

## 4. Export Pumps 

The amount of drug that reaches its intracellular targets, accounted for by the balance between uptake and export, determines its therapeutic effectiveness. The main proteins involved in the reduction of the intracellular concentration of drugs are pumps, which have in common their ability to transport their substrates against a concentration gradient; that is, they are primary active transporters able to directly utilize energy from ATP hydrolysis. The majority, but not all, of these pumps belong to the superfamily of ABC proteins. The expression of these proteins in epithelial barriers and as part of excretory mechanisms, such as the hepatobiliary system, greatly affects liver and other territories availability of many drugs [92]. Regarding cancer chemotherapy, these pumps constitute one of the major problems accounting for the lack of response to antitumour drugs [4]. 

Considering the ability to reduce drug content in liver cells the most interesting members of the superfamily of ABC proteins include four pumps located at the canalicular membrane of hepatocytes: P-glycoprotein or multidrug resistance protein (MDR1, gene symbol *ABCB1*), MRP2, BSEP, and breast cancer resistance protein (BCRP, gene symbol *ABCG2*). In addition, under certain circumstances, such as chemical stress, the expression of basolateral pumps, such as MRP1, MRP3, and MRP4 (Figure 2), is enhanced by a mechanism that involves a retrocontrol loop between the mitochondrial and nuclear genomes [93]. 

ABCB family includes the prototypic ABC protein, that is, MDR1, which is expressed in many epithelial and nonepithelial tissues, where it plays an important role in the export of a large variety of compounds, including many drugs (Table 4) [11, 94]. This glycoprotein consists of two halves of 6 transmembrane domains and a cytoplasmic nucleotide binding domain (NBD) each. 

In the liver this pump is important regarding three functions: (i) elimination of toxic compounds that may induce liver, and other organs, damage, (ii) elimination of drugs, which would greatly affect their bioavailability, and (iii) chemoresistance, when MDR1 is expressed in liver tumours [95–98].

Another member of the ABCB family is BSEP, which is believed to be the major mechanism accounting for the generation of the osmotic gradient of bile acids that determines the formation of an important fraction of bile flow [99]. Owing to the marked substrate specificity of this pump and its almost exclusive localization at the canalicular membrane of hepatocytes, regarding the ability to secrete drugs into bile its pharmacological relevance is low. However, due to its pivotal role in bile formation, its inhibition by several compounds known to be competitive inhibitors of BSEP may account for acquired cholestasis occurring under certain circumstances [100]. For some compounds, such as estrogens and progesterone derivatives the ability to inhibit BSEP from the canalicular lumen once has been secreted, presumably through other canalicular pumps, has been reported [101, 102]. 

Several members of the ABCC family are expressed in the liver. To understand their role in pharmacology it is important to distinguish between two groups of ABCC pumps. On one hand there are MRPs poorly expressed under physiological circumstances and located at the basolateral membrane, such as MRP1, MRP3-9 [11], whose role in drug handling is probably minor, but this is not well understood. On the other hand, MRP2 is highly expressed at the canalicular membrane, where this pump plays a crucial role in detoxification, mainly exporting compounds that have undergone phase II biotransformation, that is, conjugation with glutathione, glucuronate, sulphate, or taurine (Table 4). Recently, the presence of MRP2 at the nuclear envelop of hepatocytes together with conjugating enzymes has led to suggest that this may constitute a barrier to protect the nuclear content from genotoxic compounds [103].

However, the situation is very different in cancer chemotherapy, because liver tumour cells can overexpress one or several members of the ABCC family, which may enhance their ability to eliminate a large variety of drugs and hence becoming resistant to the pharmacological treatment [104]. Thus, the expression of MRP2 has been associated with a reduction in the efficacy of cisplatin-based chemotherapy of patients with hepatocellular carcinoma [105, 106]. Moreover, there is a marked overlapping in substrate specificity of MRP2 with other members of this family, such as MRP1 and MRP3. These have been shown to induce resistance to *Vinca *alkaloids, anthracyclines, camptothecins, chlorambucil, cisplatin, cyclophosphamide, irinotecan, methotrexate, paclitaxel, podophyllotoxins, and tamoxifen [107, 108].

In contrast to ABCB and ABCC families, whose members have two halves of six transmembrane domains with two NBD, ABCG members are half-transporters with a predicted topology of 6 transmembrane domains and a single NBD. These must form homo- or heterodimers to become functional pumps. BCRP is expressed in many territories including the canalicular membrane of hepatocytes [109]. This pump is involved in the secretion into bile of a large number of compounds, which include bile acids, although this physiological role has been recently evaluated as more important in placenta, whereas in liver this is probably minor as compared to that of BSEP [110]. 

Regarding substrate specificity, BCRP shares with MDR1 a marked but not complete overlapping (Table 4). Among the drugs transported by BCRP are important agents used in cancer chemotherapy, such as mitoxantrone, topotecan and methotrexate [111], nucleoside analogues, such as cladribine and clofarabine [112], 5-fluorouracil [113], oxaliplatin [114], and cisplatin [115], which account for the important role of this pump in the development of chemoresistance by many haematological and solid tumours, including liver tumours [116]. At this respect, we have recently reported that BCRP may play a role in chemoresistance induced by exposure of tumour cells to cisplatin [115]. The overexpression of BCRP may also play a role in the development of refractoriness to the pharmacological treatment of liver cancer in paediatric patients [117]. In addition to anticancer agents, BCRP is also capable of transporting nonchemotherapy drugs, including nitrofurantoin, prazosin, glyburide, and 2-amino-1-methyl-6-phenylimidazo [4,5-b]pyridine (PhIP) [111].

Beside ABC proteins, there are other active primary transporters able to export drugs that have been included among the mechanisms of chemoresistance type 1b (MOC-1b) [4]. These include P-type ATPases, such as the Menkes (ATP7A) and Wilson (ATP7B) proteins. These are copper transporters able to transport cisplatin derivatives [118]. Wilson protein is expressed at the canalicular membrane of hepatocytes where it plays an important role in copper homeostasis but may also be involved in the elimination of metal-containing drugs by normal liver as well as from liver-derived tumours [119], where this protein has been found highly expressed [120].

## 5. Chemosensitization

Since the activity of ABC proteins may reduce the efficacy of drugs when maintained levels of the active agent, either in general circulation or in tumour tissue, are required, an intense effort is being carried out to develop novel-specific reversing agents for these pumps, which can be included in the general concept of chemosensitizers [56, 121]. 

The ongoing search for compounds that act directly on the ABC transporter proteins to block their activity has led to three generations of drugs. Among the compounds used in the first generation of chemosensitizers are the calcium channel blocker verapamil and the immunomodulator cyclosporine A, which are able to inhibit MDR1 but are poor substrates of this pump. Both were able to efficiently resensitize *in vitro* MDR1-mediated drug resistant cancer cells. Although these compounds entered clinical trials, they failed to be useful for cancer patients due to the high dose required and the adverse effects of the combined treatment with verapamil [122–124] or cyclosporine A [125, 126]. In an attempt to enhance beneficial properties and reduce adverse effects a second generation of MDR1 modulators was developed. Among them the most promising compound was a cyclosporine A analogue, SDZ PSC833 or valspodar. This drug is 10- to 20-fold stronger in inhibiting MDR1 activity, but, unfortunately, side effects are also more serious. Clinical trials revealed that valspodar administration resulted in overexposing the patients to increased serum concentrations of cytotoxic drug [127–129]. Some of the third generation of chemosensitizers are LY335979 (zosuquidar), GF12918 (elacridar), and CBT-1 and XR9576 (tariquidar) [130]. These are highly effective even at nanomolar concentrations and have promising properties compared to those obtained in earlier generations. Interestingly, these ABC modulators are less toxic and they do not affect the pharmacokinetics of anticancer drugs [130]. Part of the differential advantages of these drugs is their specificity. For instance, zosuquidar is able to inhibit MDR1 with high efficacy (Ki approximately 60 nM) but has no inhibitory effect on MRPs or BCRP [131].

Chemosensitization can be also achieved by increasing the amount of drug taken up by tumour cells. In addition to the above commented possibility of reaching this aim by drug targeting, there is the option of restoring or enhance the expression or the function of carriers naturally involved in this process. The copper transporter CTR1, a major influx transporter for platinum drugs, can be used as an example to illustrate these possibilities. Thus, in a mouse model of human cervical cancer, the combined treatment of cisplatin with a copper chelator increases the availability of CTR1 to take up cisplatin, which results in enhanced cisplatin-DNA adduct levels in cancerous but not in normal tissues, impaired angiogenesis, and improved therapeutic efficacy. In addition, cisplatin is known to reduce CTR1 expression by stimulation of proteasomal degradation of this carrier, which limits the capability of tumour cells to take up this drug. Treatment with bortezomib, a proteasomal inhibitor, blocks cisplatin-induced CTR1 degradation and hence increases the abundance of transporter proteins at the plasma membrane of ovarian cancer cells as well as their capability to take up cisplatin, which results in enhanced activation of apoptosis [132].

## 6. Conclusions and Perspectives

Among the challenges of modern hepatic pharmacology are the understanding of the role of plasma membrane transporters in drug pharmacokinetics together with the influence of genetic and environmental factors in the expression and function of these transporters. This is important because drug transporters are involved in adverse effects due to drug interactions, which limit the clinical usefulness of some pharmacological combinations and affect the acceptance of novel drugs by regulatory agencies. Advances in this field will permit to develop new generation of drugs with lower risk of drug interactions but enhanced beneficial properties regarding increased hepatotropism and/or the ability to overcome transporter-mediated chemoresistance.

Another factor that is gaining interest because it may influence the role of transporters in the final effect of any pharmacological treatment is the existence of circadian rhythms in organs involved in drug disposition. Thus, mouse orthologues of OCT1 and OATPs expressed in liver (Oatp1a1, Oatp1a4, and Oatp1b2) have a maximal expression at approximately the second half of light day (2:00 p.m.), whereas no circadian fluctuations in the liver expression of Ntcp and Ent1 have been reported [133]. Regarding canalicular pumps, mRNA levels of the murine orthologues of BSEP, MRP2, and BCRP show mild or none circadian fluctuations, whereas those of rodent orthologue of MDR1 have a peak at 10:00 p.m. [133]. Whether similar rhythms also exist in humans is poorly understood, but it is known that disturbed circadian regulation, for instance due to jet-lag, shift work, and dysfunction of core clock genes, leads to changed periods of activity, sleep disorders, disturbed glucose homeostasis, enhanced risk of developing breast or colon cancer, and metabolic syndrome. Similarly, impairment of the physiological clock may also influence the circadian rhythm of the liver affecting good timing of drug administration, which can account for reduced success of the pharmacological treatment [134].

## Figures and Tables

**Table 1:** Identified potential substrates of hepatic forms of OATPs with pharmacological interest.

	OATP1B1	OATP1B3	OATP1A2
Substrates	Benzylpenicillin		
	Bile acid derivatives (BAMETS)	Bile acid derivatives (BAMETS)	Bile acid derivatives (BAMETS)
	Bilirubin and its conjugates	Bilirubin conjugates	
	Bosentan	Bosentan	
	BQ-123	BQ-123	BQ-123
	Bromosulfophthalein	Bromosulfophthalein	Bromosulfophthalein
	Caspofungin		
	Cerivastatin	Cholecystokinin-8	
	Cholic acid		Cholic acid
	DHEA sulphate	DHEA sulphate	DHEA sulphate
		Deltorphin II	Deltophorin II
		Digoxin	
	DPDPE	DPDPE	DPDPE
		Docetaxel	
	Enalapril	Enalapril	
	Estradiol 17*β*-glucuronide	Estradiol 17*β*-glucuronide	
	Estrone-3-sulphate		Estrone-3-sulphate
		Fexofenadine	Fexofenadine
	Flavopiridol	Flavopiridol	
	Fluvastatin	Fluvastatin	
	Glycocholate	Glycocholate	Glycocholate
	Irinotecan and SN-38	Irinotecan and SN-38	
			Imatinib
			Levofloxacin
	Leukotriene C4	Leukotriene C4	
	Methotrexate	Methotrexate	Methotrexate
	Microcystin-LR	Microcystin-LR	Microcystin-LR
	Olmesartan	Olmesartan	
		Ouabain	Ouabain
	Paclitaxel	Paclitaxel	
	Phalloidin	Phalloidin	
			D-Penicillamine
	Pravastatin		Pravastatin
	Prostaglandin E2		Prostaglandin E2
	Rifampicin	Rifampicin	
	Rosuvastatin	Rosuvastatin	Rosuvastatin
			Saquinavir
	Taurocholate	Taurocholate	Taurocholate
		Telmisartan	
	Thromboxane B2		
	Thyroxine	Thyroxine	Thyroxine
	Triiodothyronine	Triiodothyronine	Triiodothyronine
	Troglitazone sulphate		
	Valsartan	Valsartan	

**Table 2:** Identified potential substrates of hepatic forms of OATs, OCTs, and OCTNs with pharmacological interest.

	OAT2	OCT1	OCTN1
Substrates	*p*-Aminohippurate	Acetylcholine	L-Carnitine
	Acetylsalicylate	Acyclovir	Ergothioneine
	Allopurinol	Cimetidine	Pyrilamine
	Bumetanide	Choline	Quinidine
	Cyclic AMP	Dopamine	Quinine
	DHEA sulphate	Epinephrine	Tetraethylammonium
	Estrone-3-sulphate	Famotidine	Verapamil
	5-Fluorouracil	Ganciclovir	
	Glutarate	Histamine	
	*α*-Ketoglutarate	Imatinib	
	Methotrexate	Lamivudine	
	Ochratoxin A	Metformin	
	Paclitaxel	N-Methylnicotinamide	
	Prostaglandin E2	1-Methyl-4-phenylpyridinium	
	Prostaglandin F2*α*	Norepinephrine	
	Raloxifene	Quinine	
	Salicylate	Ranitidine	
	Tetracycline	Serotonin	
	Valproic acid	Spermidine	
	Zidovudine	Spermine	
		Tetraethylammonium	
		Zalcitabine	

**Table 3:** Identified potential substrates of hepatic forms of CNTs, ENTs, and PEPTs with pharmacological interest.

	CNT1-3	ENT1-3	PEPT1-2
Substrates		Adenine	5-Aminolevulinic acid
			Amoxicillin
			Ampicillin
	Adenosine	Adenosine	Bestatin
	Benzamide riboside		Cefadroxil
	Cladribine	Cladribine	Cefixime
	Clofarabin	Clofarabine	Ceftibuten
	Cytarabine		Cephalexin
	Cytidine	Cytidine	Cephradine
	Didanosine		Glycylsarcosine
	Fialuridine	Fialuridine	L-Kyotorphin
	Fludarabine	Fludarabine	
	5-Fluorouridine		
	Formycin		
	Gemcitabine	Gemcitabine	
		Guanine	
	Guanosine	Guanosine	
		Hypoxanthine	
	6-Mercaptopurine		
	Inosine	Inosine	
	Ribavirin	Ribavirin	
	Stavudine		
	6-Thioguanine		
	Thymidine	Thymidine	
	Tiazofurin	Tiazofurin	
	Uridine	Uridine	
	Zalcitabine		
	Zebularine	Zebularine	
	Zidovudine	Zidovudine	

**Table 4:** Identified potential substrates of canalicular forms of ABC proteins with pharmacological interest.

	MDR1	MRP2	BCRP
Substrates	Actinomycin D	Acetaminophen-glucuronide	Abacavir
	Amitriptyline	Acetaminophen-sulphate	Aflatoxin B
	Amsacrine	*p*-Aminohippurate	Albendazole sulfoxide
	Bisantrene	Arsenic-glutathione	Bile acids
	Camptothecins	Bilirubin-glucuronide	Ciprofloxacin
	Cerivastatin	BQ-123	Coumestrol
	Colchicine	Diclofenac-glucuronide	Daidzein
	Cyclosporine A	S-(2,4-Dinitrophenyl)-glutathione	Dantrolene
	Daunorubicin	Estradiol-17*β*-glucuronide	DHEA sulphate
	Digoxin	Ethacrynic acid-glutathione	Dipyridamole
	Diltiazem	Ethinylestradiol glucuronide	Edaravone sulphate
	Docetaxel	4-Hydroxynonenal-glutathione	Enrofloxacin
	Domperidone	Indinavir	Erlotinib
	Doxorubicin	Leukotriene C4	Estradiol-17*β*-glucuronide
	Erlotinib	Methotrexate	Estrone-3-sulphate
	Erythromycin	Morphine-3-glucuronide	Etoposide
	Etoposide	Ochratoxin A	Furosemide
	Fexofenadine	Oxidized/reduced glutathione	Gefitinib
	Imatinib	PhIP	Genistein
	Indinavir	Ritonavir	Glyburide
	Ivermectin	Saquinavir	Grepafloxacin
	Lapatinib	Sulfotaurolithocholic acid	Hematoporphyrin
	Loperamide	Taurocholic acid	Hoechst
	Losartan	Taurolithocholate sulphate	Hydrochlorothiazide
	Lovastatin	Vinblastine	Imatinib
	Methotrexate	Vincristine	Lamivudine
	Mitoxantrone		Lapatinib
	Nelfinavir		Methotrexate
	Ondansetron		Mitoxantrone
	Oseltamivir		Nitrofurantoin
	Paclitaxel		Norfloxacin
	Phenytoin		Ofloxacin
	Prazosin		Oxfendazole
	Quinidine		Pheophorbide A
	Ritonavir		PhIP
	Saquinavir		Prazosin
	Sparfloxacin		Resveratrol 3-sulphate
	Tamoxifen		Resveratrol di-sulphate
	Terfenadine		Riboflavin
	Tetracycline		Rosuvastatin
	(99m)Tc-Tetrofosmin		Triamterene
	Topotecan		Ulifloxacin
	Vecuronium		Zidovudine
	Verapamil		
	Vinblastine		
	Vincristine		
